# Clinical Outcomes of Cardiac Implantable Electronic Device Infections in Octogenarians: A 20-Year Retrospective Cohort Study

**DOI:** 10.3390/jcm15082996

**Published:** 2026-04-15

**Authors:** Sameer Al-Maisary, Migdat Mustafi, Gabriele Romano, Matthias Karck, Rawa Arif, Patricia Kraft, Mario Jesus Guzman-Ruvalcaba

**Affiliations:** 1 Department of Cardiothoracic Surgery, University Hospital of Saarland, Kirrberger Straße 57, 66421 Homburg, Germany; migdat.mustafi@uks.eu; 2 Department of Cardiac Surgery, Heidelberg University Hospital, 69120 Heidelberg, Germany; gabriele.romano@med.uni-heidelberg.de (G.R.); matthias.karck@med.uni-heidelberg.de (M.K.); rawa.arif@med.uni-heidelberg.de (R.A.); patricia.kraft@med.uni-heidelberg.de (P.K.); guzaman18@gmail.com (M.J.G.-R.)

**Keywords:** octogenarians, cardiac implantable electronic device (CIED), infection, lead extraction

## Abstract

**Background:** The global demographic shift towards an aging population has driven a steady, exponential increase in the utilization of cardiac implantable electronic devices (CIEDs). Consequently, device-related infectious complications have emerged as a leading cause of morbidity and healthcare expenditure. Patients in their eighth decade of life—octogenarians (aged 80–90 years)—represent an exceptionally high-risk demographic due to the compounding factors of physiological frailty, immunosenescence, and complex multi-morbidity. Despite this growing demographic, their specific clinical presentations, microbiological profiles, and procedural outcomes following infection remain poorly defined in the current literature. This study aimed to comprehensively compare the clinical characteristics, pathogen distribution, and in-hospital outcomes of CIED infections in an octogenarian cohort against a younger patient population. **Methods:** We conducted a robust retrospective cohort analysis of 383 consecutive patients treated for confirmed CIED infections at one major tertiary referral center (Heidelberg University Hospital) between January 2002 and December 2022. The cohort was stratified by age into octogenarians (*n* = 76) and a younger control group (*n* = 307). We systematically extracted and compared data regarding baseline clinical presentation, chronic comorbidities, detailed microbiological cultures (pocket, blood, and extracted leads), and definitive in-hospital outcomes, primarily mortality and length of stay. **Results:** The octogenarian cohort exhibited a significantly heavier comorbidity burden, notably higher rates of coronary artery disease (51.3% vs. 29.6%, *p* < 0.001), systemic hypertension (55.3% vs. 38.1%, *p* = 0.007), and chronic obstructive pulmonary disease (7.9% vs. 1.6%, *p* = 0.003). Furthermore, therapeutic systemic anticoagulant use was substantially more prevalent in the elderly group (60.5% vs. 45.0%, *p* = 0.015). Octogenarians presented overwhelmingly with localized generator pocket infections (73.0% vs. 30.0%, *p* < 0.001) but paradoxically also demonstrated higher rates of systemic bacteremia and sepsis (26.3% vs. 15.0%, *p* = 0.019). Microbiological analysis revealed a unique pathogen profile, with *Staphylococcus capitis* found with significantly higher frequency in the generator pockets of the elderly cohort. Remarkably, despite possessing a higher average lead burden (2.1 vs. 1.2 leads) and extreme comorbidity profiles, octogenarians demonstrated no statistically significant differences in in-hospital mortality (3.9% vs. 4.2%, *p* = 1.000) or overall length of hospital stay (14.7 vs. 17.2 days, *p* = 0.386) when compared to the younger cohort. **Conclusions:** Octogenarians suffering from CIED infections display highly distinct clinical and microbiological profiles, characterized predominantly by elevated rates of localized pocket infections, specific opportunistic pathogens, and a severe underlying comorbidity burden. Crucially, our findings indicate that with the application of modern extraction and management protocols, advanced age alone does not intrinsically correlate with increased in-hospital mortality. Future prevention and perioperative management strategies tailored to this rapidly expanding demographic must heavily prioritize the mitigation of pocket-related complications, particularly considering the high prevalence of concurrent anticoagulation therapy.

## 1. Introduction

The global demographic shift toward an aging population has driven a steady, exponential increase in the utilization of cardiac implantable electronic devices (CIEDs). This trend is directly reflected in the proportional rise in device-related infections, a complication that is particularly prevalent and concerning among elderly populations [1,2]. Consequently, these infectious complications have emerged as a leading cause of patient morbidity and significant healthcare expenditure within modern cardiology. Patients in their eighth decade of life—octogenarians, defined as those aged 80 to 90 years—represent an exceptionally high-risk demographic. This specific age cutoff was selected because it represents a unique clinical tipping point characterized by a complex intersection of physiological frailty, immunosenescence, and a dense burden of multi-morbidity. Unlike younger cohorts, these patients often possess a significantly decreased physiological reserve, making clinical decision-making regarding aggressive interventions like lead extraction exceptionally difficult. Within the current clinical landscape, these patients represent a high-risk cohort due to the interplay of these significant comorbidities and their decreased physiological reserve [3,4]. While CIED infections significantly impact clinical outcomes and healthcare costs across all age groups, the specific risk profile and long-term prognosis for octogenarians remain exceptionally complex and difficult to predict [5,6]. Existing studies indicate that older age may correlate with higher infection rates during both the initial device implants and subsequent generator replacements [7,8]. Despite the rapid growth of this vulnerable demographic, their specific clinical presentations, microbiological profiles, and procedural outcomes following a confirmed infection remain poorly defined in the current literature. Given the high morbidity associated with complex extraction procedures in the elderly, a deeper understanding of these specific risk factors is crucial for effective prevention and clinical management [9]. Octogenarians suffering from CIED infections often display highly distinct clinical and microbiological profiles compared to younger patients. These are characterized predominantly by significantly elevated rates of localized generator pocket infections and the presence of specific opportunistic pathogens, all occurring within a landscape of severe underlying chronic disease. Furthermore, this population faces specific perioperative risks related to their pharmacological profiles, most notably the high prevalence of concurrent systemic anticoagulation therapy. Future prevention and management strategies tailored to this demographic must heavily prioritize the mitigation of pocket-related complications. This study provides incremental value beyond existing literature by identifying highly distinct clinical and microbiological signatures unique to the octogenarian population. Specifically, we characterize a paradoxical divergence where elderly patients present with significantly higher rates of localized generator pocket infections while simultaneously exhibiting a higher incidence of severe systemic sepsis. Furthermore, we explore the unique prevalence of opportunistic pathogens, such as *Staphylococcus capitis*, which appear to drive infection in this demographic.

To address these gaps, this study aimed to comprehensively compare the clinical characteristics, pathogen distribution, and in-hospital outcomes of CIED infections in an octogenarian cohort against a younger patient population. We conducted a robust retrospective cohort analysis of 383 consecutive patients treated for confirmed CIED infections at a single major tertiary referral centers between January 2002 and December 2022. The cohort was stratified by age into octogenarians (*n* = 76) and a younger control group (*n* = 307). We systematically extracted and compared data regarding baseline clinical presentation, chronic comorbidities, and detailed microbiological cultures from the generator pocket, blood, and extracted leads. Finally, we evaluated definitive in-hospital outcomes, focusing primarily on mortality and the total length of hospital stay to determine if advanced age independently correlates with poorer prognosis.

## 2. Materials and Methods

### 2.1. Study Design and Study Population

This research was structured as a comprehensive, Single-center, retrospective cohort study specifically designed to elucidate and evaluate the clinical characteristics, procedural complexities, and definitive outcomes of CIED-related infections occurring within the octogenarian demographic. The clinical data were systematically extracted and aggregated from the highly detailed electronic medical records of all consecutive patients who received a formal clinical diagnosis of a CIED-related infection, and subsequently underwent surgical and medical treatment, between 1 January 2002, and 31 December 2022. The participating institution, Heidelberg University Hospital, serves as major tertiary referral center for complex cardiovascular interventions and advanced lead extraction.

To facilitate comparative statistical analysis, the extracted patient cohort was strictly categorized into two distinct groups based on chronological age at the exact time of the infection diagnosis. The primary group of interest, designated as “octogenarians,” was defined as patients aged between 80 and 90 years. The control group, designated as the “younger cohort” or “other ages,” comprised all remaining adult patients under the age of 80 who suffered from a CIED infection during the identical 20-year timeframe.

### 2.2. Data Collection and Variable Definitions

For every individual patient included in the study, a rigorous and comprehensive dataset of clinical, procedural, and microbiological variables was collected (File S1).

- **Demographics and Global Clinical Presentation:** This included absolute age, biological sex, anthropometric data (weight, height, body mass index [BMI]), and the total length of the hospital stay (measured in consecutive days from admission to discharge or death).
- **Comorbidity Burden:** A highly detailed profile of chronic medical conditions was constructed to accurately assess physiological reserve. The documented comorbidities included structurally defined coronary artery disease (CAD), systemic essential hypertension, documented prior cerebrovascular accidents (stroke), prior acute myocardial infarctions, clinically diagnosed congestive heart failure, diabetes mellitus, chronic obstructive pulmonary disease (COPD), and baseline renal functional status (specifically noting advanced renal insufficiency or the absolute requirement for active hemodialysis).
- **Pharmacological Profile:** Recognizing the critical impact of medical therapy on hemostasis and immune function, the current active use of systemic anticoagulant therapy and systemic corticosteroid regimens at the time of admission was meticulously recorded.
- **Procedural Characteristics:** The mechanical and historical aspects of the patient’s device therapy were documented. Variables included the absolute number of previous CIED-related surgical procedures (initial implants, generator changes, upgrades), the absolute total number of indwelling transvenous leads at the time of infection, the specific type of electronic device in situ (Pacemaker, Implantable Cardioverter-Defibrillator (ICD), or Cardiac Resynchronization Therapy (CRT)), the anatomical location of the generator pocket (e.g., standard pre-pectoral vs. abdominal pocket), and the presence or absence of surgical epicardial leads.

### 2.3. Infection Classification and Microbiological Protocols

To ensure diagnostic uniformity, all suspected CIED infections were rigorously classified based on a synthesis of direct clinical examination, serological markers, and advanced radiographic imaging into one of three primary categories: localized pocket infection, lead-associated endocarditis, or broad systemic endocarditis (encompassing any valvular or generalized systemic involvement). The presence of severe systemic manifestations, explicitly including documented bloodstream bacteremia and clinical sepsis, was recorded for each case.

Comprehensive microbiological data were gathered from multiple infectious sites to establish the pathogen profile. This included the isolation and definitive identification of pathogens from standard positive aerobic and anaerobic blood cultures, direct swabbing and tissue biopsy of the infected device pocket during surgical exploration, and the rigorous culturing of the extracted lead tips immediately following their transvenous removal.

### 2.4. Clinical Outcomes

The primary clinical outcome evaluated in this study was absolute in-hospital mortality, representing the most definitive and severe endpoint following a CIED infection and its subsequent management. Secondary endpoints were designed to assess healthcare utilization and pathophysiological correlations; these included the total length of hospital stay (LOS) and the specific statistical correlation between baseline patient comorbidities and the exact genus and species of the isolated infecting pathogen.

### 2.5. Statistical Analysis

All statistical analyses were executed with precision to uncover significant differences between the age cohorts. Continuous mathematical variables were systematically tested for normality of distribution using Kolmogorov–Smirnov test. Normally distributed continuous variables were expressed as the mean ± standard deviation (SD), whereas non-normally distributed data were expressed as the median alongside the interquartile range (IQR). Categorical variables were uniformly expressed as absolute frequencies and their corresponding percentages. We used the R from RStudio software, Version 4.5.3. for statistical analysis

To determine statistical significance in the comparisons between the octogenarian cohort and the younger cohort, we employed Student’s *t*-test or the non-parametric Mann–Whitney U test for continuous clinical data, depending on distribution parameters. For the analysis of categorical clinical data, we utilized the Chi-square test, applying Fisher’s exact test in instances where expected cell counts in the contingency tables fell below standard thresholds. Across all utilized statistical models, a two-tailed *p*-value of <0.05 was strictly considered to denote statistical significance.

## 3. Results

### 3.1. Baseline Demographics and Study Population

Following the rigorous application of our inclusion criteria, the final, consolidated study cohort comprised a total of 383 unique patients who were definitively diagnosed with, and treated for, CIED-related infections over the 20-year study period. Upon stratification by age at the time of infection, 76 patients (representing 19.8% of the total cohort) were classified as octogenarians (aged 80–90 years), while the remaining 307 patients (80.2%) were categorized into the “Other ages” younger control group.

The mean chronological age of the specialized octogenarian group was 83.3 ± 2.9 years (median 82.5), providing a stark demographic contrast to the mean age of 63.4 ± 12.2 years (median 66.0) observed in the younger cohort (*p* < 0.001). Despite this two-decade gap in average age, demographic baseline uniformity was maintained in other core physical parameters. Specifically, there were no statistically significant differences between the two study groups regarding biological sex distribution (72.4% male in octogenarians vs. 75.9% in younger patients, *p* = 0.524) or overall body mass index (BMI) (27.1 ± 3.9 kg/m^2^ vs. 27.8 ± 5.3 kg/m^2^, *p* = 0.431). Height and weight parameters were also statistically comparable across both cohorts (Table 1).

### 3.2. Complex Comorbidity Profiles

A deep analysis of the patients’ underlying medical histories revealed that octogenarians presented to the hospital with a profoundly different, and significantly heavier, burden of specific chronic comorbidities. The absolute prevalence of structural coronary artery disease was strikingly elevated, affecting over half (51.3%) of all octogenarians, compared to only 29.6% in the younger patient group (*p* < 0.001). Similarly, systemic essential hypertension was documented in 55.3% of the elderly cohort versus 38.1% of the younger cohort, achieving statistical significance (*p* = 0.007). Furthermore, chronic obstructive pulmonary disease (COPD), a condition known to complicate surgical anesthesia and wound healing, was vastly more frequent in the octogenarians (7.9% vs. 1.6%, *p* = 0.003).

Interestingly, a highly significant inverse relationship was observed regarding congestive heart failure. A clinical diagnosis of heart failure was significantly less common within the octogenarian group (17.1%) when compared to the younger cohort, where it was prevalent in over a third of patients (37.1%, *p* < 0.001). When evaluating other major systemic diseases, including diabetes mellitus (31.6% vs. 22.5%, *p* = 0.098), varying degrees of renal insufficiency (31.6% vs. 25.4%, *p* = 0.276), dependence on hemodialysis (2.6% vs. 4.9%, *p* = 0.542), prior myocardial infarction, or a documented prior history of stroke, no statistically significant differences were observed between the age brackets (Table 2).

### 3.3. Procedural History and Device Characteristics

The fundamental distribution of the implanted electronic device types differed significantly as a function of patient age. The octogenarian population was substantially more likely to be managed with standard permanent pacemakers (61.8% vs. 42.0%, *p* = 0.002) and Cardiac Resynchronization Therapy (CRT) devices (18.4% vs. 8.5%, *p* = 0.011). Conversely, they were significantly less likely to have received Implantable Cardioverter-Defibrillators (ICDs) compared to their younger counterparts (18.4% vs. 39.1%, *p* < 0.001).

Beyond the device type, the structural complexity of the indwelling hardware was also remarkably different. Octogenarians carried a significantly higher overall burden of intravascular hardware, defined by a higher mean number of transvenous leads (2.1 ± 1.0 leads vs. 1.2 ± 1.2 leads, *p* < 0.001). Interestingly, despite this higher lead burden, the absolute number of previous surgical CIED procedures (such as initial implants or generator changes) was statistically comparable between both groups (2.0 ± 1.2 vs. 1.9 ± 1.8, *p* = 0.072). Crucially for perioperative risk assessment, the baseline utilization of systemic anticoagulant therapy was significantly higher in the vulnerable octogenarian group (60.5% vs. 45.0%, *p* = 0.015). Systemic corticosteroid use was low and comparable between groups (3.9% vs. 2.3%, *p* = 0.423) (Table 3).

### 3.4. Clinical Manifestation of Infection

The anatomical and clinical presentation of the CIED infections showcased a profound and distinct age-related divergence. Octogenarians presented with a vastly higher, statistically significant rate of localized generator pocket infections (73.0% vs. 30.0%, *p* < 0.001). Corroborating this clinical finding, they were also significantly more likely to yield definitively positive microbial cultures directly swabbed or biopsied from the device pocket itself (43.4% vs. 26.4%, *p* = 0.004).

While one might expect this localized propensity to spare them from systemic issues, the data revealed a complex reality. There was no statistically significant difference between the age groups concerning the incidence of deep intravascular lead endocarditis (28.9% vs. 21.2%, *p* = 0.148) or broader systemic endocarditis involving any structural valves (17.1% vs. 14.0%, *p* = 0.494). However, octogenarians paradoxically exhibited a significantly higher overall incidence of severe systemic manifestations, specifically documented bacteremia and clinical sepsis (26.3% vs. 15.0%, *p* = 0.019). This was further validated by a higher rate of definitively positive systemic blood cultures (28.9% vs. 17.9%, *p* = 0.032) (Table 4).

### 3.5. Detailed Microbiological Ecosystem

The exhaustive microbiological profiling of the CIED infections uncovered highly distinct, age-dependent ecological patterns across the various anatomical culture sites. As illustrated in the comparative analysis matrices, octogenarians demonstrated a notably higher localized proliferation of specific opportunistic pathogens within the device pockets.

**Figure 1:**
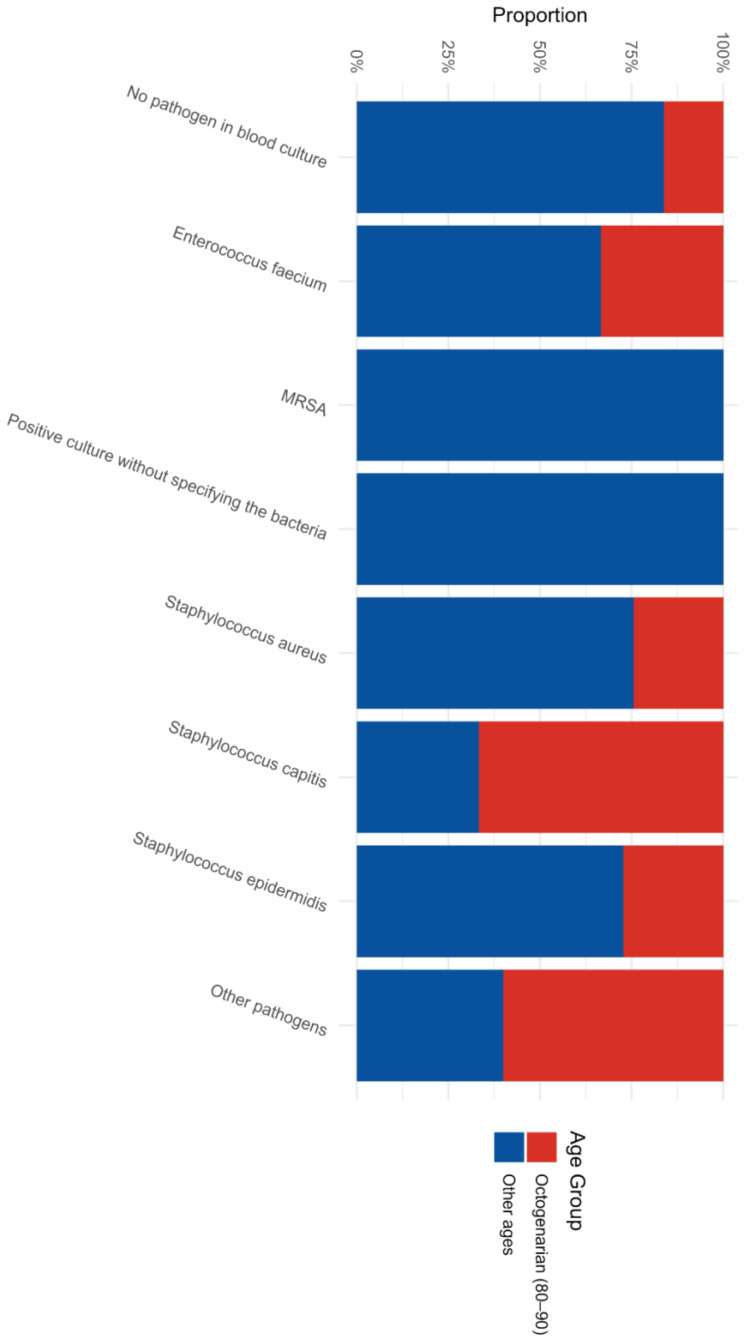
Distribution of Pathogens in Device Pocket Cultures by Age Group.

Most prominently, *Staphylococcus capitis*, a known coagulase-negative skin commensal, was predominantly and uniquely identified as a major driver of pocket infections within the octogenarian group (Figure 1). In stark contrast, the younger patient demographic demonstrated a much higher relative prevalence of highly virulent *Staphylococcus aureus* and classical *Staphylococcus epidermidis* in their local pocket cultures.

**Figure 2:**
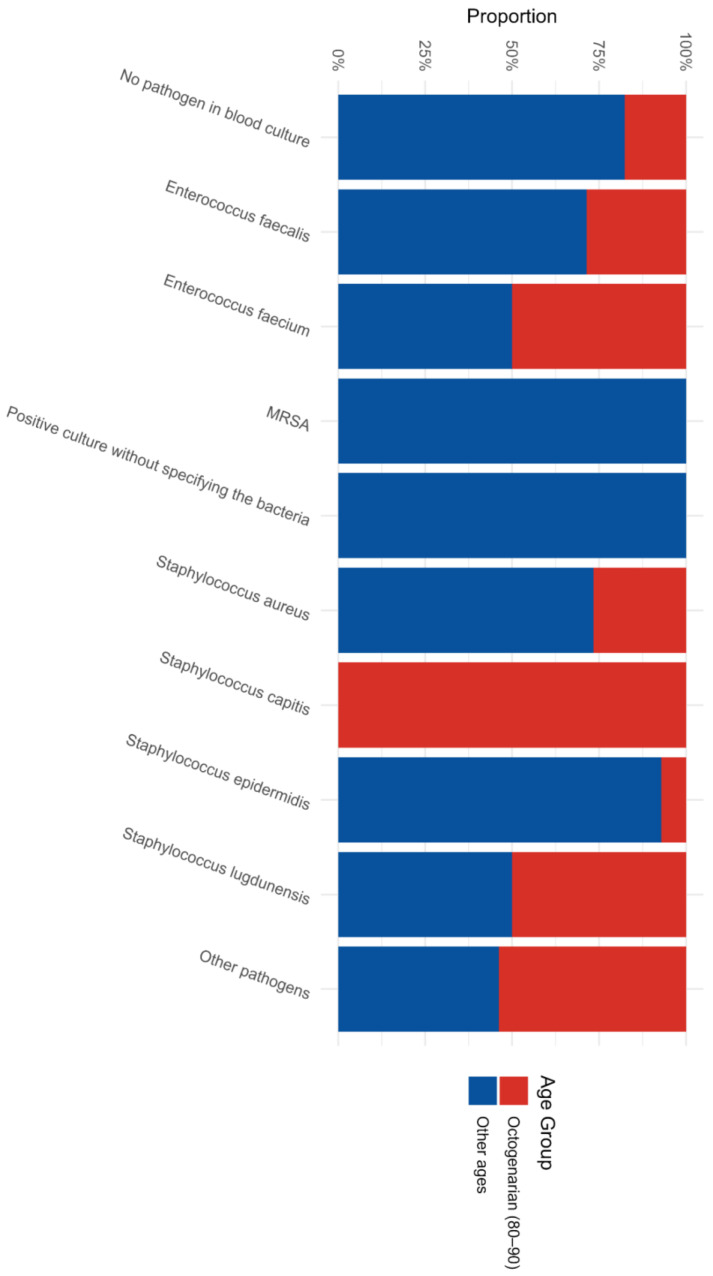
Distribution of Pathogens in Blood Cultures by Age Group.

When examining the systemic bloodstream manifestations, the pathogen split remained evident (Figure 2). Octogenarians remarkably accounted for approximately half of all systemic *Enterococcus faecium* and *Staphylococcus lugdunensis* infections successfully identified via blood cultures in the entire study. While the younger patients comprised the absolute entirety of Methicillin-Resistant *Staphylococcus aureus* (MRSA) and other unspecified aggressive bacterial positive cultures, the sheer volume of bacteremia in the elderly meant that octogenarians represented nearly 30% of the entire study cohort presenting with positive blood cultures.

**Figure 3:**
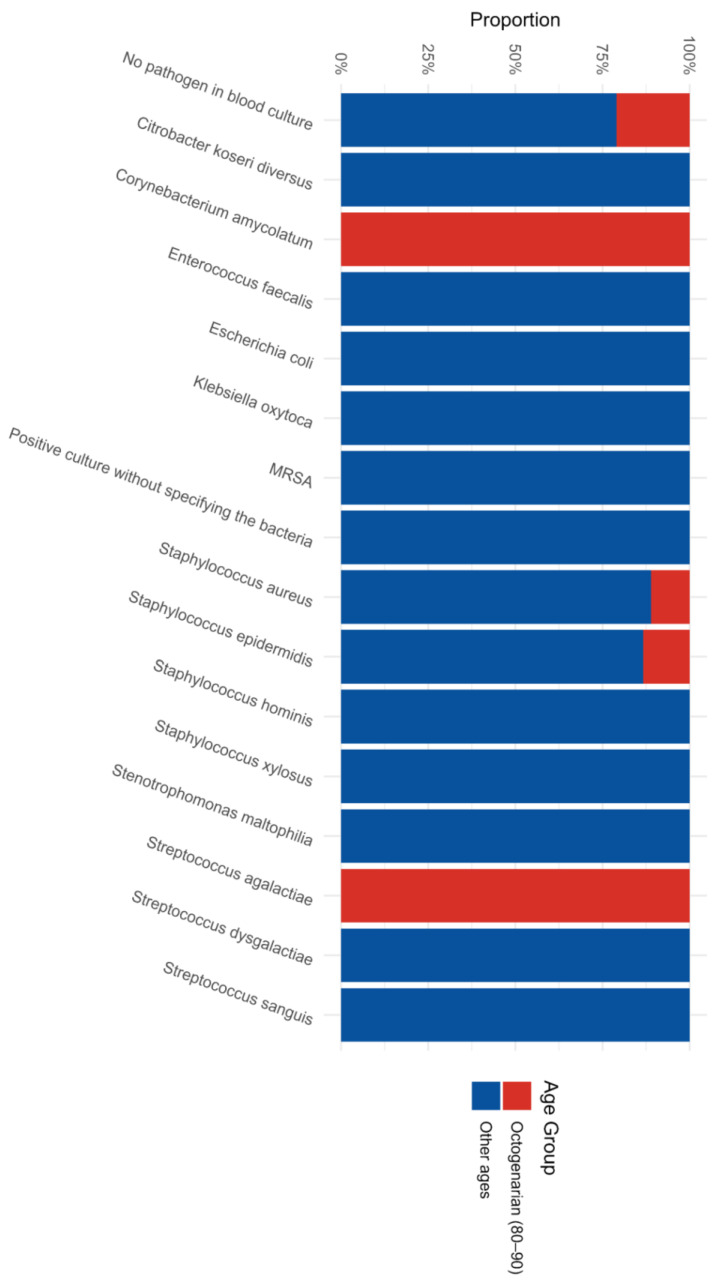
Distribution of Pathogens on Extracted Leads by Age Group.

The recovery of viable pathogens directly from the tips of the extracted transvenous leads was comparatively less frequent overall in the octogenarian group (7.9%) than in the younger cohort (14.0%) (*p* = 0.153) (Figure 3). However, the composition of these lead-based biofilms was unique. Certain rare, insidious pathogens, specifically *Corynebacterium amycolatum* and *Streptococcus agalactiae*, were isolated exclusively from the extracted leads of octogenarian patients. Conversely, a significantly broader and more aggressive diversity of Gram-negative bacteria (e.g., *Escherichia coli*, *Klebsiella oxytoca*) and varying *Staphylococcus* species were routinely isolated from the leads of the younger group. These figures comprehensively highlight that while ubiquitous staphylococcal species inevitably remain the prevalent driving force of CIED infections, octogenarians harbor a specific subset of opportunistic pathogens, particularly driving localized pocket morbidity.

### 3.6. Definitive In-Hospital Outcomes

Perhaps the most striking finding of the entire analysis pertained to the primary outcome of survival. Despite the patients’ advanced extreme age, their vastly more complex multi-lead device systems, and their significantly heavier burden of chronic physiological comorbidities, the raw in-hospital mortality rate was not significantly different between the two cohorts. Octogenarians experienced a 3.9% in-hospital mortality rate, virtually identical to the 4.2% rate observed in the significantly younger, theoretically more resilient control group (*p* = 1.000). Furthermore, this equality in outcome extended to healthcare resource utilization; the total length of hospital stay required to manage and clear the infection did not differ statistically between the age groups (14.7 ± 18.7 days for octogenarians vs. 17.2 ± 30.3 days for younger patients, *p* = 0.386).

## 4. Discussion

The management of CIED infections in octogenarians presents a unique clinical challenge, as this population is characterized by increased physiological frailty and a complex burden of chronic comorbidities [1,2,10]. Our findings indicate that while octogenarians (aged 80–90) represent approximately 19.8% of the infection cohort, their clinical profile and infection manifestations differ significantly from younger patients. This mirrors global epidemiological trends where advanced age and the proliferation of complex devices have led to a steady rise in CIED-related hospitalizations and healthcare expenditures [11,12,13].

The diagnostic challenges in managing CIED infections in the elderly are compounded by the high prevalence of multi-morbidity and competing pathologies. Our findings underscore that while clinical and laboratory data are foundational, a multimodality imaging approach is essential for accurate diagnosis and for defining the extent of the infection. This is particularly critical in octogenarians, who possess a higher likelihood of concurrent malignancies. In such patients, right-sided intracardiac masses or vegetations attached to leads may not always represent infective endocarditis; they may instead reflect cardiac tumors or non-bacterial thrombotic endocarditis. Consequently, imaging must go beyond simple detection. Echocardiography remains the first-line modality and often provides initial clues regarding the nature of a cardiac mass, but it should be integrated into a broader diagnostic framework—including advanced imaging when necessary—to support differential diagnosis and guide surgical or interventional strategy [14]. By employing this comprehensive imaging strategy, clinicians can better distinguish between infectious processes and masquerading conditions, ensuring that aggressive interventions like lead extraction are reserved for confirmed cases of CIED infection

Octogenarians in this study exhibited a significantly higher prevalence of coronary artery disease (51.3%), hypertension (55.3%), and COPD (7.9%) compared to younger cohorts [1,7,15]. This aligns with broader evidence suggesting that older age correlates with a higher risk of infection during both primary implants and subsequent generator replacements [8,16,17]. Interestingly, heart failure was significantly less common in the octogenarian group (17.1%) compared to other ages (37.1%), a phenomenon that may reflect a survival bias or distinct indications for device implantation in the very elderly [3,4].

A critical risk factor identified in our cohort was the higher use of anticoagulant therapy in octogenarians (60.5%). Anticoagulation has been historically linked to increased pocket hematoma formation, which is a well-established precursor to device infection [5,18]. The BRUISE CONTROL trial demonstrated that while specific management strategies can mitigate this risk, the overall incidence of bleeding remains a significant concern in elderly patients requiring multiple antithrombotic agents [18,19].

The clinical presentation of infection showed a distinct age-related shift. Octogenarians were significantly more likely to present with localized pocket infections (73.0% vs. 30.0%) and positive pocket cultures (43.4%). This localized manifestation may be due to the increased frequency of generator replacements in this age group, which primarily involves the device pocket [10,20]. Conversely, while systemic endocarditis was not more frequent, the higher incidence of bacteremia (26.3%) in octogenarians highlights a risk for rapid systemic decompensation if localized symptoms are not aggressively addressed [6,9].

A significant and paradoxical finding of this study is the divergence between the anatomical and clinical presentation of infections in octogenarians. While this elderly cohort presented with a vastly higher rate of localized generator pocket infections compared to younger patients (73.0% vs. 30.0%), they simultaneously demonstrated a significantly higher incidence of severe systemic manifestations, including documented bacteremia and clinical sepsis (26.3% vs. 15.0%). This suggests that although the initial infectious insult in octogenarians is often confined to the device pocket—likely due to the high frequency of generator replacements and the high prevalence of anticoagulation-related hematomas—the physiological frailty and immunosenescence characteristic of this age group may facilitate rapid systemic seeding. Consequently, what appears to be a localized complication in an octogenarian carries a disproportionately high risk for systemic decompensation, necessitating aggressive early intervention and standardized extraction protocols to prevent adverse outcomes.

Microbiologically, the prevalence of *Staphylococcus capitis* and Corynebacterium in the elderly cohort highlights the role of skin-derived commensals in device-related infections [21,22]. While *Staphylococcus aureus* remains the most virulent pathogen across all age groups, coagulase-negative staphylococci are increasingly recognized for their ability to form resilient biofilms on device hardware, particularly in the immunocompromised or elderly patient [15,21,23].

Despite the perceived high risk of intervention, our study found that transvenous lead extraction (TLE) is both safe and effective in octogenarians. Modern extraction techniques, including laser-assisted technology, have shown high success rates with low procedural mortality in elderly cohorts [18,20,24]. In our study, in-hospital mortality was not significantly different between octogenarians (3.9%) and the younger cohort (4.2%), supporting the consensus that advanced age alone should not be a contraindication for complete hardware removal [9,19,23].

Finally, prevention remains paramount. Large-scale clinical trials, such as WRAP-IT and PADIT, have emphasized the importance of antibacterial envelopes and intensified prophylactic antibiotic regimens in high-risk groups [11,25]. Tailoring these prevention strategies to the elderly is essential for improving long-term outcomes in this growing demographic [15,19].

## 5. Conclusions

This study demonstrates that octogenarians with CIED infections present with a distinct clinical and microbiological profile, characterized by higher rates of pocket infections, bacteremia, and specific comorbidities like hypertension and COPD. However, advanced age in itself was not associated with increased in-hospital mortality or prolonged hospitalization. Given the high morbidity of these infections, prevention strategies should be tailored to address the high prevalence of anticoagulation and pocket-related risks in the elderly.

### Study Limitation

This study has several limitations, primarily its retrospective, single-center design conducted at a tertiary referral center, which may limit the generalizability of the findings to other healthcare settings. The 20-year study period (2002–2022) introduces potential documentation bias and reflects significant shifts in CIED technology and procedural protocols over time. Additionally, the strict definition of octogenarians as patients aged 80–90 years excludes those over 90, a group that may present distinct clinical risks. The observed lower prevalence of heart failure in the elderly cohort compared to younger patients suggests a possible survival bias, where only relatively healthier octogenarians were candidates for device therapy or survived to present with infections. Finally, the reliance on electronic medical records across two decades may result in inconsistencies in the documentation of chronic comorbidities. Also, the lack of long-term follow-up is a constraint of this retrospective design.

## Figures and Tables

**Table 1:** Patient Demographics and Anthropometric Data.

Variable	Overall (*n* = 383)	Octogenarian (80–90) (*n* = 76)	Other Ages (*n* = 307)	*p* Value
Age, years	67.4 ± 13.6; 70.0 (59.0–77.0)	83.3 ± 2.9; 82.5 (81.0–86.0)	63.4 ± 12.2; 66.0 (57.5–73.0)	<0.001
Male sex	288 (75.2%)	55 (72.4%)	233 (75.9%)	0.524
Female sex	95 (24.8%)	21 (27.6%)	74 (24.1%)	0.524
Body-mass index, kg/m^2^	27.6 ± 5.1; 27.3 (24.5–30.5)	27.1 ± 3.9; 26.4 (25.0–29.1)	27.8 ± 5.3; 27.5 (24.3–30.8)	0.431
Weight, kg	73.3 ± 31.4; 80.0 (65.0–90.0)	73.3 ± 25.9; 76.0 (67.0–87.7)	73.3 ± 32.7; 80.0 (65.0–91.0)	0.424
Height, cm	151.3 ± 56.8; 172.0 (163.0–177.8)	157.4 ± 47.3; 170.0 (164.0–175.5)	149.8 ± 58.8; 172.0 (162.0–178.0)	0.429

**Table 2:** Comorbidities and Pharmacotherapy.

Variable	Overall (*n* = 383)	Octogenarian (80–90) (*n* = 76)	Other Ages (*n* = 307)	*p* Value
Coronary artery disease	130 (33.9%)	39 (51.3%)	91 (29.6%)	<0.001
Hypertension	159 (41.5%)	42 (55.3%)	117 (38.1%)	0.007
Heart failure	127 (33.2%)	13 (17.1%)	114 (37.1%)	<0.001
COPD	11 (2.9%)	6 (7.9%)	5 (1.6%)	0.003
Diabetes mellitus	93 (24.3%)	24 (31.6%)	69 (22.5%)	0.098
Renal insufficiency	102 (26.6%)	24 (31.6%)	78 (25.4%)	0.276
Dialysis	17 (4.4%)	2 (2.6%)	15 (4.9%)	0.542
Prior myocardial infarction	77 (20.1%)	16 (21.1%)	61 (19.9%)	0.818
Prior stroke	30 (7.8%)	9 (11.8%)	21 (6.8%)	0.146
Anticoagulant therapy	184 (48.0%)	46 (60.5%)	138 (45.0%)	0.015
Systemic corticosteroids	10 (2.6%)	3 (3.9%)	7 (2.3%)	0.423

**Table 3:** Device and Procedural Characteristics.

Variable	Overall (*n* = 383)	Octogenarian (80–90) (*n* = 76)	Other Ages (*n* = 307)	*p* Value
Pacemaker	176 (46.0%)	47 (61.8%)	129 (42.0%)	0.002
ICD	134 (35.0%)	14 (18.4%)	120 (39.1%)	<0.001
CRT	40 (10.4%)	14 (18.4%)	26 (8.5%)	0.011
Number of leads	1.3 ± 1.2; 1.0 (0.0–2.0)	2.1 ± 1.0; 2.0 (2.0–2.0)	1.2 ± 1.2; 1.0 (0.0–2.0)	<0.001
Previous CIED procedures	1.9 ± 1.7; 2.0 (1.0–3.0)	2.0 ± 1.2; 2.0 (1.0–3.0)	1.9 ± 1.8; 2.0 (1.0–3.0)	0.072

ICD: Implantable Cardioverter-Defibrillator; CRT: Cardiac Resynchronization Therapy.

**Table 4:** Infection Characteristics and Clinical Outcomes.

Variable	Overall (*n* = 383)	Octogenarian (80–90) (*n* = 76)	Other Ages (*n* = 307)	*p* Value
Pocket infection	146 (38.3%)	54 (73.0%)	92 (30.0%)	<0.001
Bacteremia/sepsis	66 (17.2%)	20 (26.3%)	46 (15.0%)	0.019
Positive blood culture	77 (20.1%)	22 (28.9%)	55 (17.9%)	0.032
Positive pathogen in pocket	114 (29.8%)	33 (43.4%)	81 (26.4%)	0.004
Positive pathogen on leads	49 (12.8%)	6 (7.9%)	43 (14.0%)	0.153
Endocarditis (any)	56 (14.6%)	13 (17.1%)	43 (14.0%)	0.494
Lead endocarditis	87 (22.7%)	22 (28.9%)	65 (21.2%)	0.148
In-hospital mortality	16 (4.2%)	3 (3.9%)	13 (4.2%)	1
Length of stay, days	16.7 ± 28.4; 8.0 (4.0–17.0)	14.7 ± 18.7; 9.0 (6.0–18.2)	17.2 ± 30.3; 8.0 (4.0–16.5)	0.386

## Data Availability

All underlying data supporting the conclusions of this manuscript are contained comprehensively within the Supplementary Material provided with this publication.

## Supplementary Materials

The following supporting information can be downloaded at: https://www.mdpi.com/article/10.3390/jcm15082996/s1, File S1. Study flowchart.
