# Supplementary material for: Clinical Outcomes of Cardiac Implantable Electronic Device Infections in Octogenarians: A 20-Year Retrospective Cohort Study
Source: J Clin Med. 2026 Apr 15;15(8):2996. doi: 10.3390/jcm15082996 (PMC13117004; doi:10.3390/jcm15082996)
Supplement: Supplementary file 1 [file jcm-15-02996-s001.zip › jcm-4243875-supplementary.pdf]

Study Flow Chart: Patient Selection and Stratification

Total CIED-Infection Population Scanned *(January 2002 – December 2022)* **n = 383** consecutive patients treated at Heidelberg University Hospital

|

|

Screening & Inclusion Criteria Applied * Formal clinical diagnosis of CIED-related infection

- Underwent both surgical and medical treatment (standardized extraction protocols)
- Complete electronic medical records available for analysis

|

|

Final Analyzed Cohort n = 383 unique patients

|

|--|

| |

Group A: Octogenarians Group B: Control Group ("Other Ages") n = 76 (19.8%) n = 307 (80.2%)

(Aged 80–90 years) (Aged < 80 years)

| |

| |

Data Extraction Categories (n=383)

- **Clinical Presentation:** Localized pocket, lead endocarditis, or systemic involvement
- **Comorbidity Profile:** CAD, Hypertension, COPD, Renal status, Heart Failure
- **Microbiology:** Pocket swabs, Lead cultures, and Blood cultures
- **Device History:** Type of CIED, lead burden, and previous procedures

|

|

Outcome Analysis Primary Endpoint: In-hospital mortality

Secondary Endpoints: Length of stay (LOS) and pathogen-comorbidity correlations
